# Supplementary material for: Exosomal miR92a Promotes Cytarabine Resistance in Myelodysplastic Syndromes by Activating Wnt/β-catenin Signal Pathway
Source: Biomolecules. 2022 Oct 9;12(10):1448. doi: 10.3390/biom12101448 (PMC9599242; doi:10.3390/biom12101448)
Supplement: Supplementary file 1 [file biomolecules-12-01448-s001.zip › biomolecules-1930892-supplementary.pdf]

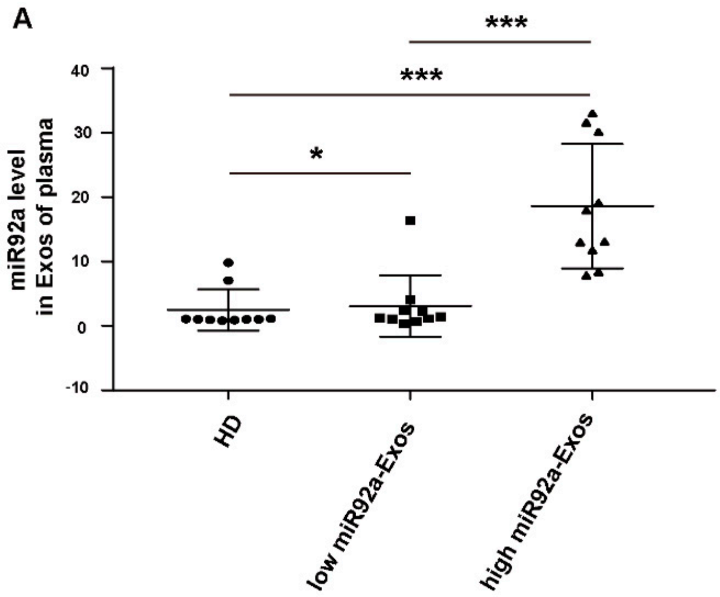

Figure S1. (A) miR92a level in Exos derived from plasma of HD (Exos-HD), MDS/AML patients (Exos-miR92a high and Exos-miR92a low).

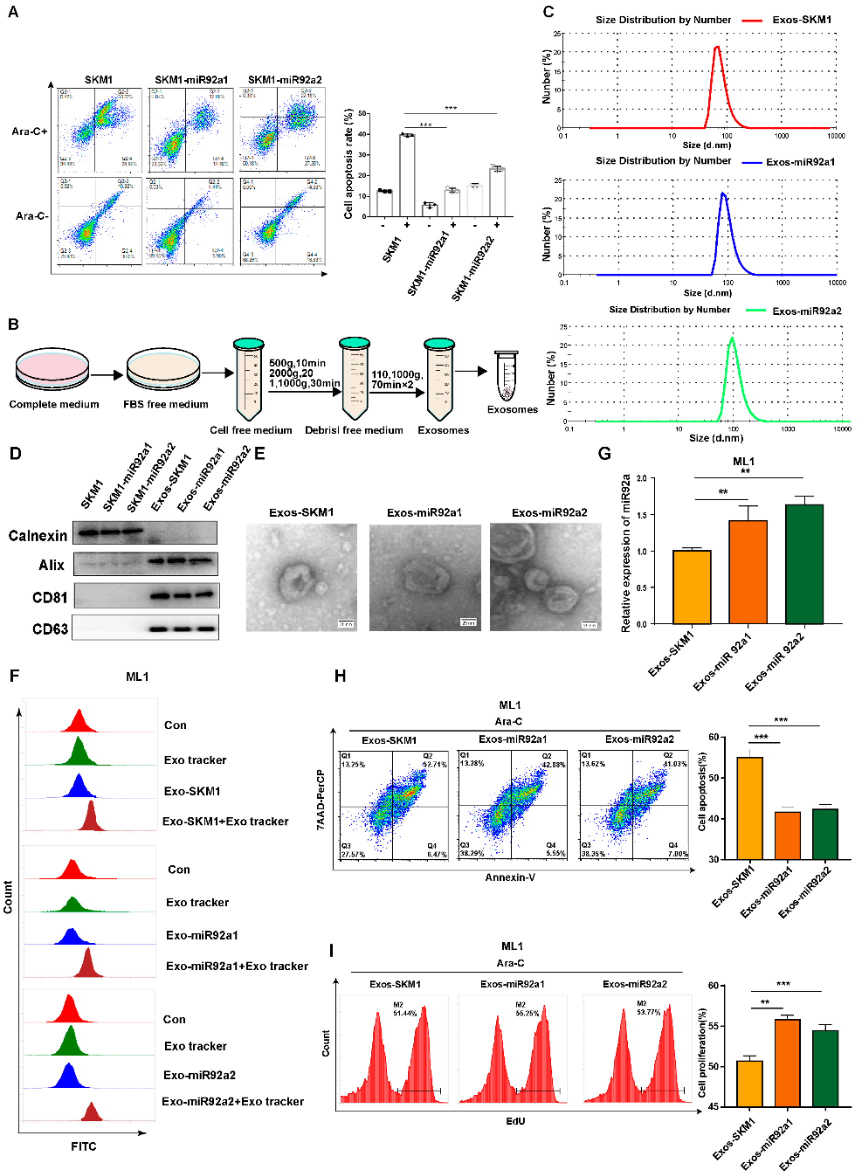

Figure S2. Exosomal miR92a increased resistance to Ara-C of ML1 cells. (A) Flow cytometry analysis of cell apoptosis of SKM1, SKM1-miR92a1 and SKM1-miR92a2 under 2 μM Ara-C treatment for 48 h. (B) The scheme of exosome isolation from culture medium of SKM1, SKM1-miR92a1 and SKM1-miR92a2. (C) Particle size of Exos by NTA. (D) Detection of Exo markers by Western blotting. (E) Examination of Exo morphology by TEM. (F) Uptake of Exos-SKM1 and Exos-miR92a1/2 in SKM1 analyzed by flow cytometry. (G) miR92a level in ML1 treated with Exos-SKM1, Exos -miR92a1/2 for 48 h. (H-I) Flow cytometry analysis of cell apoptosis (H) and proliferation (I) of ML1 treated by 2 μM Ara-C and 10 μg Exos-SKM1 or Exos -miR92a1/2 for 48 h.

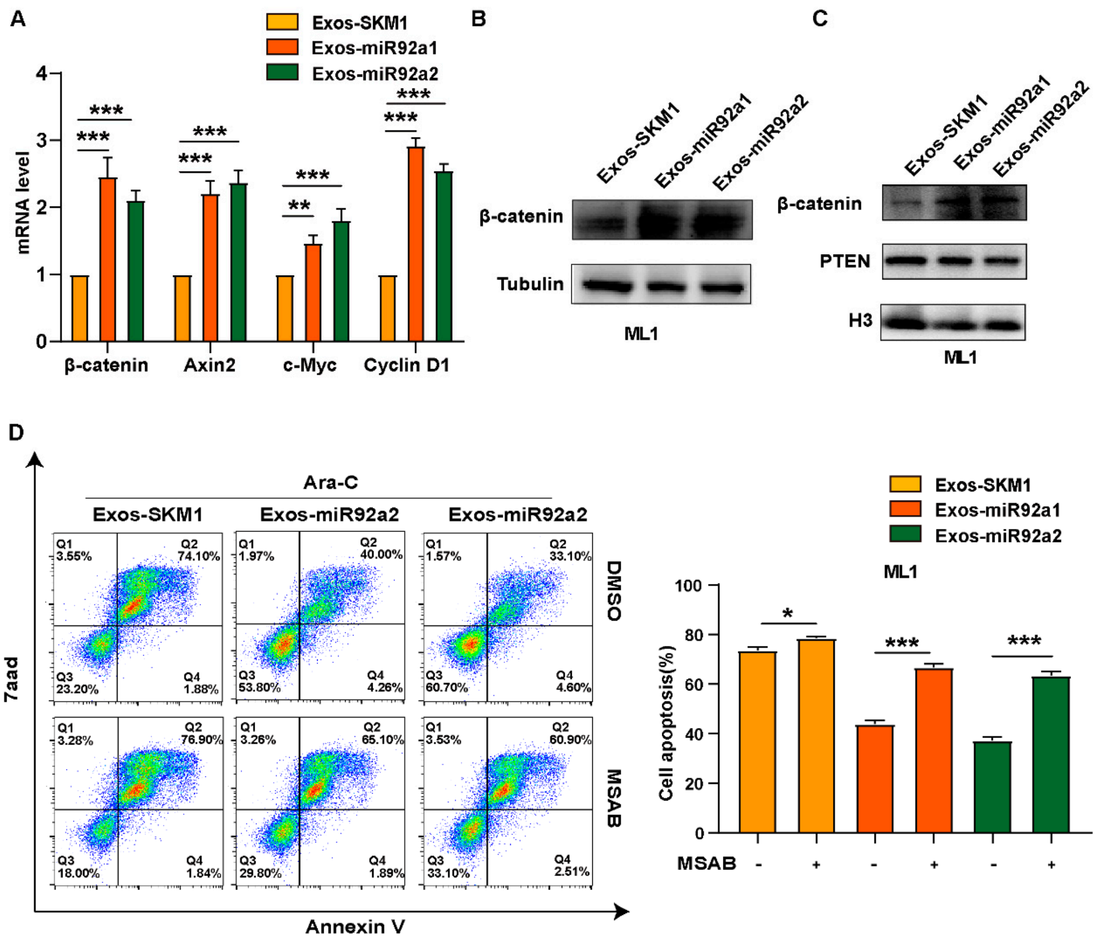

Figure S3. miR92a regulates ML1 cell growth via activating Wnt/β-catenin signal. (A) Expression of β-catenin, Axin2, c-Myc and Cyclin D1 in Exos-SKM1, Exos-miR92a1, Exos-miR92a2 treated ML1 cells analyzed by qRT-PCR. (B) Expression of β-catenin in ML1 treated with Exos-SKM1, Exos-miR92a1, Exos-miR92a2 by Western Blotting. (C) PTEN and β-catenin in the nucleus of ML1 treated with Exos-SKM1, Exos-miR92a1, Exos-miR92a2. (D) Apoptosis of Exos-SKM1, Exos-miR92a1 or Exos-miR92a2 treated ML1 under 2.5 μM MSAB and 2 μM Ara-C treatment for 48 h respectively.

Table S1. Patient list.

diagnosis	age (yr)	gender	miR92a	WBC（*10^9/L）	PLT（*10^9/L）	IPSS
AML	63	M	high	171.09	69	2
AML from MDS	16	M	high	27.12	4	1.5
MDS	56	M	high	6.07	122	0
AML	53	M	high	21.1	62	0.5
MDS	66	F	high	11.11	8	1
AML	55	F	high	14.27	25	1.5
MDS	77	M	high	2.46	32	2
MDS	62	F	high	3.22	5	1.5
AML	47	F	high	123	5	> 2.5
AML	83	M	high	28.1	62	1
MDS	71	F	low	7.22	653	1.5
AML	66	M	low	23.66	30	1
AML	64	F	low	68.34	30	0
AML	42	F	low	66.7	145	1
AML	78	F	low	3.41	87	1.5
MDS	32	F	low	2.52	13	1
MDS	73	F	low			1
MDS	49	F	low	3.52	54	2
MDS	77	F	low	2.03	36	1
AML	55	F	low	99.16	49	0
AML from MDS	28	M	low	9.72	19	0
HD	45	F	-	-	-	-
HD	35	F	-	-	-	-
HD	27	F	-	-	-	-
HD	61	F	-	-	-	-
HD	28	F	-	-	-	-
HD	31	F	-	-	-	-

Table S2. Primer list.

Primer	Sequence (5' to 3')
miR92a1F	GCGAGCACAGAATTAATACGAC
miR92a1R	GCGAGCACAGATTACTTGTGT
miR92a2F	TTGTCCCGGCCTGTGGAAA
miR92a2R	GAAGGTGAAGGTCGGAGTC
PTEN WT F	GTAATTCTAGGCGATCGCTGAGTTTCTGGAATATTTT
PTEN WT R	TTTATTGCGGCCAGCTCAGTGCATAGCATT
PTEN MUT 1 F	GTAATTCTAGGCGATCGCTCGAGCTCCTCTTGGAACATG
PTEN MUT 1 R	TTATGAACATAGTATAAAAAATTTCTAGGAACTAA
PTEN MUT 2 F	GAAATTTTTATACATGTTCATAACGATGGCT
PTEN MUT 2 R	TTTATTGCGGCCAGCGGCCGCTTTAGTATATATATATTCTA TATGAAACAGGTTGAAC
U6 F	CAGCACATATACTAAAATTGGAACG
U6 R	ACGAATTTGCGTGTCATCC
miR92a	TATTGCACTTGTCCCGGCCTGT
GAPDH F	GAAGGTGAAGGTCGGAGTC
GAPDH R	GAAGATGGTGATGGGATTTC
β-catenin F	AGCCGACACCAAGAAGCA
β-catenin R	GGCCACCCATCTCATGTTC
SMAD4 F	CACTATGAGCGGGTTGTC
SMAD4 R	GGTGCTGGTGGCGTTA
ZEB2 F	CAAGAGGCGCAAACAAGCC
ZEB2 R	GGTTGGCAATACCGTCATCC
KLF4 F	CAAGTCCCGCCGCTCCATTACCAA
KLF4 R	CCACAGCCGTCCCAGTCACAGTGG
FGF2 F	TGCTGGCTTCTGTGAGTAGTG
FGF2 R	GCCCAGTTCGTTTCAGTGCCA
PTEN F	TCCCAGACATGACAGCCATC
PTEN R	TGCTTTGAATCCAAAAACCTTACT
Axin 2 F	ACTGCCCACACGATAAGGAG
Axin 2 R	CTGGCTATGTCTTTGGACCA
c-Myc F	GCTGCTTAGACGCTGGATTT
c-Myc R	CACCGAGTCGTAGTCGAGGT
cyclin D1 F	CCATCCAGTGGAGGTTTGTC
cyclin D1 R	AGCGTATCGTAGGAGTGGGA
